# 4-(3-Alkyl/benzyl-guanidino)benzenesulfonamides as selective carbonic anhydrase VII inhibitors

**DOI:** 10.1080/14756366.2022.2080816

**Published:** 2022-05-29

**Authors:** Morteza Abdoli, Simone Giovannuzzi, Claudiu T. Supuran, Raivis Žalubovskis

**Affiliations:** a Faculty of Materials Science and Applied Chemistry, Institute of Technology of Organic Chemistry, Riga Technical University, Riga, Latvia; b Neurofarba Department, Universita Degli Studi di Firenze, Florence, Italy; c Latvian Institute of Organic Synthesis, Riga, Latvia

**Keywords:** Neurological diseases, neuropathic pain, carbonic anhydrase isozyme VII, sulfonamides, guanidine-benzenesulfonamides

## Abstract

The treatment of chronic neuropathic pain remains one of the most challenging of all neurological diseases and very much an art. There exists no consensus for the optimal management of this condition at the moment. Gaining inspiration from recent studies which pointed out the involvement of brain-associated carbonic anhydrase (CA, EC 4.2.1.1) isoform VII in the pathology of various neurodegenerative diseases, which highlighted the relationship between selective inhibition of this isozyme and relieve of neuropathic pain, herein we report the synthesis and CA VII inhibitory activity of novel 4-(3-alkyl/benzyl-guanidino)benzenesulfonamides. Ten benzyl-substituted and five alkyl-substituted 4-guanidinobenzenesulfonamide derivatives were obtained, some of which (**7c**, **7h**, **7m** and **7o**) exhibited satisfactory selectivity towards CA VII over CA I and II, with K_I_-s in the subnanomolar range and good selectivity indexes for inhibiting the target versus the off-target isoforms.

## 1. Introduction

As its name suggests, carbonic anhydrases (CAs, EC 4.2.1.1) are enzymes which catalyse the reversible hydration/dehydration of carbon dioxide (CO_2_) to bicarbonate (HCO_3_^−^) and protons (H^+^)^1^. These enzymes are constitutively produced in all tissues, organs and cells and comprise 15 different isoforms (CA I, II, III, VA, VB, VI, VII, VIII, IX, X, XI, XII, XIII, XIV and XV) in humans, according to their subcellular localisation^2^. There is increasing evidence that CAs play a key role in a variety of diseases, including edoema, epilepsy, cancer, glaucoma, haemolytic anaemia, obesity, sterility and other disorders^3^. Hence, isoform-selective targeting hCAs is an important approach for discovery and development of selective, effective and safe novel drugs^4^.

Primary sulfonamides were discovered as CA inhibitors (CAIs) in the ‘40s of the last century, and majority of the drugs launched in the next decades (diuretics, antiepileptics, or antiglaucoma agents) belonged to this class of compounds or to their isosteres such as the sulfamates and sulfamides^5–9^. A major pitfall of the first generation of CAIs was their lack of isoform selectivity, keeping in mind that in humans are present at least 12 catalytically active and three acatalytic isoforms^5–13^. In last decade a discovery was made and the new generation of CAIs belonging to coumarins and sulfocoumarins and their bioisosteres showed significant isoform-selective inhibition profiles, as demonstrated in a number of studies^14–32^. This is principally due to the fact that these compounds possess a distinct inhibition mechanism compared to the sulfonamides, which coordinate to the zinc ion from the CA active site as anions^5–13^. Recently so-called tail approach also has proven to give considerable inhibition selectivity among CA isoforms in case of primary sulfonamides^31^^,^^32^. This approach was chosen also for this study.

Neuropathic pain is a neurological disorder caused by a lesion or disease affecting the peripheral or central nervous system^33^. Often, patients with chronic neuropathic pain experience severe and unrelenting pain; thus sometimes opioid analgesics are prescribed to relieve pain^34^. Anticonvulsant drugs acting at calcium channels (e.g. pregabalin and gabapentin) and antidepressant agents (e.g. duloxetine) are the first-line options for management of this pain^35^^,^^36^. However, their efficacies are not high, and also associated with several side effects. Therefore, undoubtedly there is an unmet medical need to discover a new pharmacological class for the treatment of neuropathic pain.

**Figure 1.**
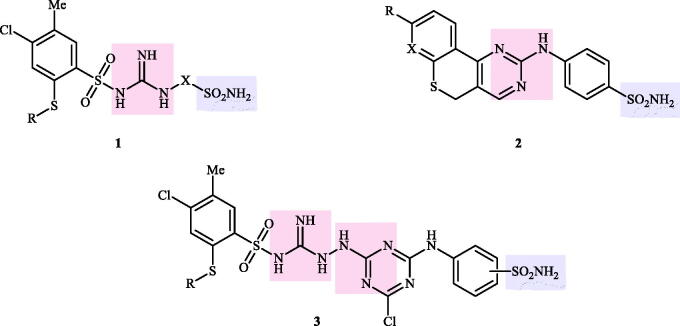
Selected examples of the guanidine-containing sulfonamide CAIs.

**Figure 2.**
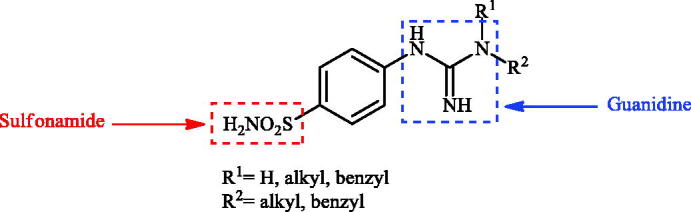
General structure of 4-(3-alkyl/benzyl-guanidino)benzenesulfonamides discussed in the paper.

Although the mechanisms of neuropathic pain for big extend remain unclear, recent studies have highlighted the involvement of the brain-associated CA VII isoform in the pathology of this syndrome^37^. Thereby, isoform-selective CAVII inhibitors are recognised as promising agents for management of neuropathic pain. Needless to say that primary sulfonamides (R-SO_2_NH_2_) are the main class of CA inhibitors and logically utmost studies on the inhibition of CA VII are relying on the use of sulfonamide-based compounds^38^. Intriguingly, recent works by one of our groups indicated that the incorporation of guanidine moiety into sulfonamide-containing compounds resulting in CA inhibitors with enhanced efficiency and selectivity (Figure 1)^39–41^. Keeping these interesting facts in mind and in connection with our works on the field of CA inhibitors^16^^,^^19^^,^^27^^,^^42^, we decided to synthesis a series of novel 4–(3-alkyl/benzyl-guanidino)benzenesulfonamides and investigate their inhibitory activity against CA VII (Figure 2).

## 2. Experimental section

### 2.1. Chemistry

Reagents, starting materials and solvents were obtained from commercial sources and used as received. Thin-layer chromatography was performed on silica gel, spots were visualised with UV light (254 and 365 nm). NMR spectra were recorded on Bruker 300 spectrometer with chemical shifts values (*δ*) in ppm relative to TMS using the residual DMSO-d_6_ signal (^1^H 2.50; ^13^C 39.52). High-resolution mass spectra (HRMS) were recorded on a mass spectrometer with a Q-TOF micro mass analyser using the ESI technique.

### 2.2. Synthesis

#### 2.2.1. 4-Thioureidobenzenesulfonamide (5)


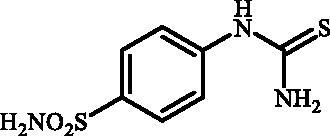


To a solution of 4-aminobenzensulfonamide (30 g, 174.3 mmol) in 3.5 M HCl (180 ml), which was heated at 70 °C and cooled to room temperature, KSCN (16.94 g, 174.3 mmol) was added and the mixture was refluxed for 3 h. After cooling to room temperature, the reaction mixture was diluted with ice-cold water. Solids were collected by filtration, washed with water and air dried to afford **5** (12.1 g, 31%) as white powder.

^1^H NMR (300 MHz, DMSO-d_6_) *δ* = 7.32 (s, 2H), 7.69 (d, 2H, *J =* 8.6 Hz), 7.77 (d, 2H, *J =* 8.6 Hz), 10.02 (s, 1H) ppm ^13^C NMR (75 MHz, DMSO-d_6_) *δ* = 122.8, 127.3, 139.8, 143.9, 182.8 ppm MS (ESI) [M + H]^+^: *m*/*z* 232.0.

#### 2.2.2. Methyl (4-sulfamoylphenyl)carbamimidothioate (6)


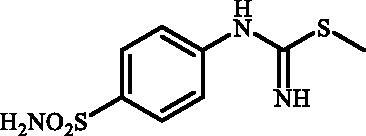


To a solution of 4-thioureidobenzenesulfonamide (**5**) (10.0 g, 43.28 mmol) in DMF (100 ml), MeI (2.69 ml, 43.28 mmol) at room temperature was added and the mixture was heated at 40 °C for 2.5 h. After cooling to room temperature water (150 ml) was added and the mixture was extracted with EtOAc (3 × 50 ml). Organic layer was washed with aq. sat. NaHCO_3_ (2 × 50 ml) and aq. sat. NH_4_Cl (50 ml), and dried over Na_2_SO_4_. Solvent evaporation in vacuum afforded **6** (7.43 g, 70%) as white powder.

^1^H NMR (300 MHz, DMSO-d_6_) *δ* = 2.37 (s, 3H), 6.63 (s, 2H), 6.94 (s, 2H), 7.22 (s, 2H), 7.71 (d, 2H, *J =* 8.4 Hz) ppm ^13^C NMR (75 MHz, DMSO-d_6_) *δ* = 14.2, 122.8, 127.7, 138.0, 153.9, 157.0 ppm HRMS (ESI) [M + H]^+^: *m*/*z* calcd for (C_8_H_12_N_3_O_2_S_2_) 246.0371. Found 246.0372.

#### 2.2.3. 4-(3-Benzyl-guanidino)benzenesulfonamide (7a)


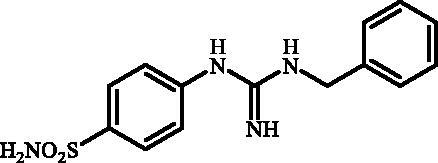


To a solution of **6** (300 mg, 1.22 mmol) in DMSO (8 ml), benzylamine (1.066 ml, 9.76 mmol) was added and the reaction mixture was stirred in sealed tube at 130 °C for 2 h. After cooling to room temperature water (40 ml) was added and the mixture was extracted with EtOAc (3 × 20 ml). The organic phase was dried over Na_2_SO_4_ and the solvent was removed under reduced pressure. The residue was dissolved in *i*PrOH (5 ml) under gentle heating and product was precipitated by addition of hexanes (40 ml). Precipitate was collected by filtration and dried under vacuum to afford the **7a** as white solids (187 mg, 50%).

^1^H NMR (300 MHz, DMSO-d_6 _+D_2_O) *δ* = 4.39 (2H, s), 6.93 (d, 2H, *J =* 7.8 Hz), 7.29 (s, 1H), 7.37 (s, 4H), 7.65 (d, 2H, *J =* 7.8 Hz) ppm ^13^C NMR (75 MHz, DMSO-d_6 _+D_2_O) *δ* = 44.4, 123.1, 127.2, 127.3, 127.7, 128.7, 135.1, 140.7, 152.6, 154.9 ppm HRMS (ESI) [M + H]^+^: *m*/*z* calcd for (C_14_H_17_N_4_O_2_S) 305.1072. Found 305.1078.

#### 2.2.4. 4-(3-(4-Methoxybenzyl)guanidino)benzenesulfonamide (7b)


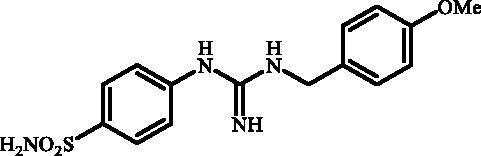


To a solution of **6** (300 mg, 1.22 mmol) in DMSO (8 ml), (4-methoxyphenyl)methanamine (1.275 ml, 9.76 mmol) was added and the reaction mixture was stirred in sealed tube at 130 °C for 2 h. After cooling to room temperature water (40 ml) was added and mixture was extracted with EtOAc (3 × 20 ml). The organic phase was dried over Na_2_SO_4_. Solvent evaporation under reduced pressure afforded **7b** (305 mg, 74%) as white powder.

^1^H NMR (300 MHz, DMSO-d_6_ + D_2_O) *δ* = 3.74 (s, 3H), 4.30 (s, 2H), 6.90 (s, 2H), 6.93 (s, 2H), 7.29 (d, 2H, *J =* 8.4 Hz), 7.64 (d, 2H, *J =* 8.4 Hz) ppm ^13 ^C NMR (75 MHz, DMSO-d_6 _+D_2_O) *δ* = 44.7, 56.3, 114.9, 123.8, 128.1, 129.9, 133.5, 135.7, 153.5, 155.9, 159.4 ppm HRMS (ESI) [M + H]^+^: *m*/*z* calcd for (C_15_H_19_N_4_O_3_S) 335.1178. Found 335.1180.

#### 2.2.5. 4-(3-(4-Fluorobenzyl)guanidino)benzenesulfonamide (7c)


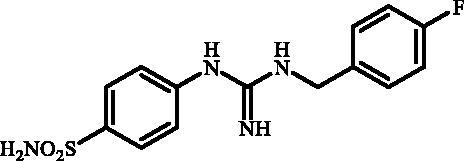


To a solution of **6** (300 mg, 1.22 mmol) in DMSO (8 ml), (4-fluorophenyl)methanamine (1.110 ml, 9.76 mmol) was added and the reaction mixture was stirred in sealed tube at 130 °C for 2 h. After cooling to room temperature water (40 ml) was added and mixture was extracted with EtOAc (3 × 20 ml). The organic phase was dried over Na_2_SO_4_ and the solvent was removed under reduced pressure. The residue was dissolved in *i*PrOH (5 ml) under gentle warming and product was precipitated by addition of hexanes (40 ml). Precipitate was collected by filtration and dried in vacuum to afford **7c** (354 mg, 90%) as white powder.

^1^H NMR (300 MHz, DMSO-d_6 _+D_2_O) *δ* = 4.36 (s, 2H), 6.92 (d, 2H, *J =* 8.1 Hz), 7.15–7.43 (m, 4H), 7.64 (d, 2H, *J =* 8.1 Hz) ppm ^13^C NMR (75 MHz, DMSO-d_6 _+D_2_O) *δ* = 44.5, 116.2 (d, *J =* 20.9 Hz), 123.8, 128.1, 130.5 (d, *J =* 7.6 Hz), 136.1, 137.7, 153.3, 155.2, 162.4 (d, *J =* 240.6 Hz) ppm HRMS (ESI) [M + H]^+^: *m*/*z* calcd for (C_14_H_16_N_4_O_2_FS) 323.0978. Found 323.0990.

#### 2.2.6. 4-(3-(3-Fluorobenzyl)guanidino)benzenesulfonamide (7d)


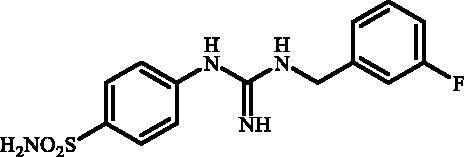


To a solution of **6** (300 mg, 1.22 mmol) in DMSO (8 ml), (3-fluorophenyl)methanamine (1.113 ml, 9.76 mmol) was added and the reaction mixture was stirred in sealed tube at 130 °C for 2 h. After cooling to room temperature water (40 ml) was added and mixture was extracted with EtOAc (3 × 20 ml). The organic phase was dried over Na_2_SO_4_ and the solvent was removed under reduced pressure. The residue was dissolved in *i*PrOH (5 ml) under gentle warming and product was precipitated by addition of hexanes (40 ml). Precipitate was collected by filtration and dried in vacuum to afford **7d** (286 mg, 72%) as white powder.

^1^H NMR (300 MHz, DMSO-d_6 _+D_2_O) *δ* = 4.42 (s, 2H), 6.93 (d, 2H, *J =* 6.9 Hz), 7.07–7.41 (m, 4H), 7.65 (d, 2H, *J =* 6.9 Hz) ppm ^13^C NMR (75 MHz, DMSO-d_6 _+D_2_O) *δ* = 40.6, 114.6 (d, *J =* 30.7 Hz), 114.9 (d, *J =* 30.7 Hz), 123.8, 124.4, 128.1, 131.4 (d, *J =* 8.1 Hz), 136.2, 144.8 (d, *J =* 6.9 Hz), 153.3, 155.0, 163.5 (d, *J =* 241.5 Hz) ppm HRMS (ESI) [M + H]^+^: *m*/*z* calcd for (C_14_H_16_N_4_O_2_SF) 323.0978. Found 323.0986.

#### 2.2.7. 4-(3-(2-Fluorobenzyl)guanidino)benzenesulfonamide (7e)


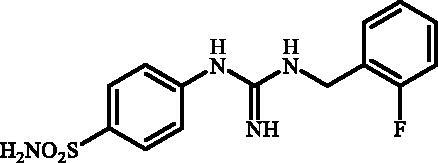


To a solution of **6** (300 mg, 1.22 mmol) in DMSO (8 ml), (2-fluorophenyl)methanamine (1.110 ml, 9.76 mmol) was added and the reaction mixture was stirred in sealed tube at 130 °C for 2 h. After cooling to room temperature water (40 ml) was added and mixture was extracted with EtOAc (3 × 20 ml). The organic phase was dried over Na_2_SO_4_ and the solvent was removed under reduced pressure. The residue was dissolved in *i*PrOH (5 ml) under gentle warming and product was precipitated by addition of hexanes (40 ml). Precipitate was collected by filtration and dried in vacuum to afford **7e** (193 mg, 49%) as white powder.

^1^H NMR (300 MHz, DMS-O-d_6 _+D_2_O) *δ* = 4.43 (s, 2H), 6.92 (d, 2H, *J =* 8.3 Hz), 7.127.50 (m, 4H), 7.64 (d, 2H, *J =* 8.3 Hz) ppm ^13^C NMR (75 MHz, DMSO-d_6 _+D_2_O) *δ* = 39.1, 116.2 (d, *J =* 21.0 Hz), 123.7, 125.5 (d, *J =* 3.2 Hz), 128.0 (d, *J =* 6.6 Hz), 128.2, 129.9 (d, *J =* 8.19 Hz), 130.7 (d, *J =* 4.6 Hz), 136.0, 153.1, 155.2, 160.3 (d, *J =* 242.4 Hz) ppm HRMS (ESI) [M + H]^+^: *m*/*z* calcd for (C_14_H_16_N_4_O_2_FS) 323.0978. Found 323.0992.

#### 2.2.8. 4-(3-(3-Methylbenzyl)guanidino)benzenesulfonamide (7f)


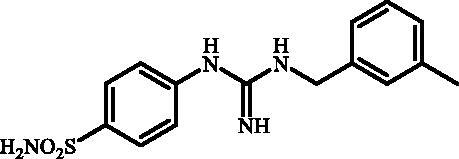


To a solution of **6** (300 mg, 1.22 mmol) in DMSO (8 ml), *m*-tolylmethanamine (1.224 ml, 9.76 mmol) was added and the reaction mixture was stirred in sealed tube at 130 °C for 2 h. After cooling to room temperature water (40 ml) was added and mixture was extracted with EtOAc (3 × 20 ml). The organic phase was dried over Na_2_SO_4_ and the solvent was removed under reduced pressure. The residue was dissolved in *i*PrOH (5 ml) under gentle warming and product was precipitated by addition of hexanes (40 ml). Precipitate was collected by filtration and dried under vacuum to afford **7f** (252 mg, 65%) as white powder.

^1^H NMR (300 MHz, DMSO-d_6 _+D_2_O) *δ* = 2.33 (s, 3H), 4.36 (s, 2H), 6.94 (d, 2H, *J =* 7.6 Hz), 7.10–7.26 (m, 4H), 7.65 (d, 2H, *J =* 7.6 Hz) ppm ^13^C NMR (75 MHz, DMSO-d_6 _+D_2_O) *δ* = 22.3, 45.2, 123.8, 125.6, 128.0, 128.6, 129.2, 129.4, 136.0, 138.6, 141.3, 153.4, 155.4 ppm HRMS (ESI) [M + H]^+^: *m*/*z* calcd for (C_15_H_19_N_4_O_2_S) 319.1229. Found 319.1241.

#### 2.2.9. 4-(3-(2-Methylbenzyl)guanidino)benzenesulfonamide (7g)


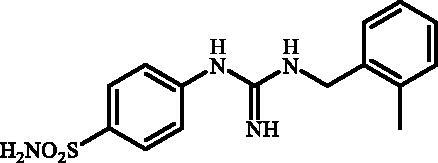


To a solution of **6** (300 mg, 1.22 mmol) in DMSO (8 ml), *o*-tolylmethanamine (1.210 ml, 9.76 mmol) was added and the reaction mixture was stirred in sealed tube at 130 °C for 2 h. After cooling to room temperature water (40 ml) was added and mixture was extracted with EtOAc (3 × 20 ml). The organic phase was dried over Na_2_SO_4_ and the solvent was removed under reduced pressure. The residue was dissolved in *i*PrOH (5 ml) under gentle warming and product was precipitated by addition of hexanes (40 ml). Precipitate was collected by filtration and dried in vacuum to afford **7g** (315 mg, 81%) as white powder.

^1^H NMR (300 MHz, DMSO-d_6 _+D_2_O) *δ* = 2.32 (s, 3H), 4.36 (s, 2H), 6.94 (d, 2H, *J =* 8.3 Hz), 7.16–7.33 (m, 4 H), 7.65 (d, 2H, *J =* 8.3 Hz) ppm ^13 ^C NMR (75 MHz, DMSO-d_6 _+D_2_O) *δ* = 19.8, 43.5, 123.9, 127.1, 128.1, 128.1, 128.8, 131.3, 136.1, 137.0, 139.0, 153.5, 155.3 ppm HRMS (ESI) [M + H]^+^: *m*/*z* calcd for (C_15_H_19_N_4_O_2_S) 319.1229. Found 319.1237.

#### 2.2.10. 4-(3-(1-Phenylethyl)guanidino)benzenesulfonamide (7h)


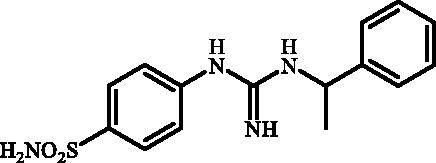


To a solution of **6** (300 mg, 1.22 mmol) in DMSO (8 ml), 1-phenylethanamine (1.258 ml, 9.76 mmol) was added and the reaction mixture was stirred in sealed tube at 130 °C for 2 h. After cooling to room temperature water (40 ml) was added and mixture was extracted with EtOAc (3 × 20 ml). The organic phase was dried over Na_2_SO_4_ and the solvent was removed under reduced pressure. The residue was dissolved in *i*PrOH (5 ml) under gentle warming and product was precipitated by addition of hexanes (40 ml). The precipitate was collected by filtration and dried in vacuum to afford **7h** (232 mg, 59%) as white powder.

^1^H NMR (300 MHz, DMSO-d_6 _+D_2_O) *δ* = 1.41 (d, 3H, *J =* 5.9 Hz), 4.97 (q, 1H, *J* = 5.9 Hz), 6.85 (d, 2H, *J* = 7.6 Hz), 7.25 (s, 1H), 7.38 (app s, 4H), 7.62 (d, 2H, *J =* 7.6 Hz) ppm ^13 ^C NMR (75 MHz, DMSO-d_6 _+D_2_O) *δ* = 24.5, 50.6, 123.8, 127.2, 127.8, 128.1, 129.6, 135.9, 146.8, 152.7, 155.5 ppm HRMS (ESI) [M + H]^+^: *m*/*z* calcd for (C_15_H_19_N_4_O_2_S) 319.1229. Found 319.1225.

#### 2.2.11. 4-(3-Methyl-3-(3-methylbenzyl)guanidino)benzenesulfonamide (7i)


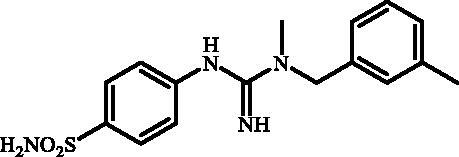


To a solution of **6** (300 mg, 1.22 mmol) in DMSO (8 ml), *N*-methyl-1-(*m-*tolyl)methanamine (1.466 ml, 9.76 mmol) was added and the reaction mixture was stirred in sealed tube at 130 °C for 2 h. After cooling to room temperature water (40 ml) was added and mixture was extracted with EtOAc (3 × 20 ml). The organic phase was dried over Na_2_SO_4_ and the solvent was removed under reduced pressure. The residual solid was washed with *i*PrOH (20 ml) and dried in vacuum to afford **7i** (292 mg, 72%) as white powder.

^1^H NMR (300 MHz, DMSO-d_6 _+D_2_O) *δ* = 2.33 (s, 3H), 2.88 (s, 3H), 4.59 (s, 2H), 6.90 (d, 2H, *J =* 8.4 Hz), 7.09-7.29 (m, 4H), 7.67 (d, 2H, *J =* 8.4 Hz) ppm ^13^C NMR (75 MHz, DMSO-d_6 _+D_2_O) *δ* = 22.4, 36.2, 53.2, 123.7, 125.6, 128.2, 128.8, 129.1, 129.6, 135.5, 138.8, 139.9, 154.5, 156.4 ppm HRMS (ESI) [M + H]^+^: *m*/*z* calcd for (C_16_H_21_N_4_O_2_S) 333.1385. Found 333.1395.

#### 2.2.12. 4-(3,3-Dibenzyl-guanidino)benzenesulfonamide (7j)


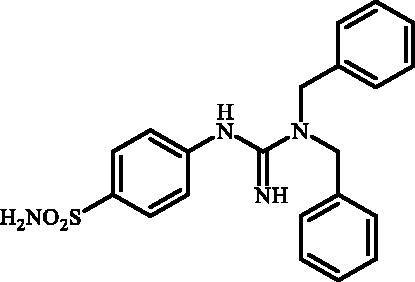


To a solution of **6** (300 mg, 1.22 mmol) in DMSO (8 ml), dibenzylamine (1.876 ml, 9.76 mmol) was added and the reaction mixture was stirred in sealed tube at 130 °C for 2 h. After cooling to room temperature water (40 ml) was added and mixture was extracted with EtOAc (3 × 20 ml). The organic phase was dried over Na_2_SO_4_ and the solvent was removed under reduced pressure. The residue was dissolved in *i*PrOH (5 ml) under gentle warming and product was precipitated by addition of hexanes (40 ml). The precipitate was collected by filtration and dried in vacuum to afford **7j** (279 mg, 58%) as white powder.

^1^H NMR (300 MHz, DMSO-d_6 _+D_2_O) *δ* = 4.58 (s, 4H), 6.91 (dd, 2H, *J =* 6.8, 1.6 Hz), 7.29–7.40 (m, 10H), 7.66 (dd, 2H, *J* = 6.8, 1.6 Hz) ppm ^13^C NMR (75 MHz, DMSO-d_6 _+D_2_O) *δ* = 50.7, 123.7, 128.2, 128.4, 129.7, 135.7, 139.7, 154.17, 156.06 ppm HRMS (ESI) [M + H]^+^: *m*/*z* calcd for (C_21_H_23_N_4_O_2_S) 395.1542. Found 395.1553.

#### 2.2.13. 4-(3-(2-Phenoxyethyl)guanidino)benzenesulfonamide (7k)


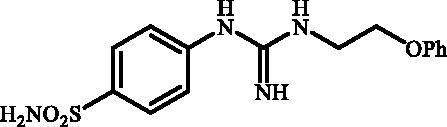


To a solution of **6** (300 mg, 1.22 mmol) in DMSO (8 ml), 2-phenoxyethanamine (1.277 ml, 9.76 mmol) was added and the reaction mixture was stirred in sealed tube at 130 °C for 6 h. After cooling to room temperature water (40 ml) was added and mixture was extracted with EtOAc (3 × 20 ml). The organic phase was dried over Na_2_SO_4_ and the solvent was removed under reduced pressure. The residue was dissolved in *i*PrOH (5 ml) under gentle warming and product was precipitated by addition of hexanes (40 ml). The precipitate was collected by filtration and dried under vacuum to afford **7k** (118 mg, 29%) as orange powder.

^1^H NMR (300 MHz, DMSO-d_6_+D_2_O) *δ* = 3.48–3.56 (m, 2H), 4.04-4.11 (m, 2H), 6.85–7.01 (m, 5H), 7.31 (q, 2H, *J =* 7.4 Hz), 7.65 (d, 2H, *J =* 8.4 Hz) ppm ^13^C NMR (75 MHz, DMSO-d_6_+D_2_O) *δ* = 41.2, 67.7, 115.6, 115.7, 121.9, 123.9, 128.1, 130.8, 136.2, 153.5, 159.7 ppm HRMS (ESI) [M + H]^+^: *m*/*z* calcd for (C_15_H_19_N_4_O_3_S) 335.1178. Found 335.1193.

#### 2.2.14. Synthesis of 4-(3-octylguanidino)benzenesulfonamide (7l)


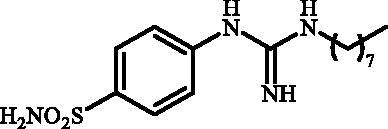


To a solution of **6** (300 mg, 1.22 mmol) in DMSO (8 ml), octan-1-amine (1.613 ml, 9.76 mmol) was added and the reaction mixture was stirred in sealed tube at 130 °C for 2 h. After cooling to room temperature water (40 ml) was added and mixture was extracted with EtOAc (3 × 20 ml). The organic phase was dried over Na_2_SO_4_ and the solvent was removed under reduced pressure. The residue was dissolved in *i*PrOH (5 ml) under gentle warming and product was precipitated by addition of hexanes (40 ml). The precipitate was collected by filtration and dried in vacuum to afford **7l** (338 mg, 85%) as white powder.

^1^H NMR (300 MHz, DMSO-d_6_+D_2_O) *δ* = 0.82–0.90 (m, 3H), 1.28 (br. s 10H), 1.42–1.50 (m, 2H), 3.11 (t, 2H, *J =* 6.7 Hz), 6.88 (d, 2H, *J =* 8.4 Hz), 7.61 (d, 2H, *J =* 8.4 Hz) ppm ^13^C NMR (75 MHz, DMSO-d_6 _+D_2_O) *δ* = 15.3, 23.5, 27.8, 30.0, 30.1, 30.5, 32.6, 41.7, 124.0, 128.1, 135.7, 153.8, 156.1 ppm HRMS (ESI) [M + H]^+^: *m*/*z* calcd for (C_15_H_27_N_4_O_2_S) 327.1855. Found 327.1867.

#### 2.2.15. 4–(3-Dodecylguanidino)benzenesulfonamide (7m)


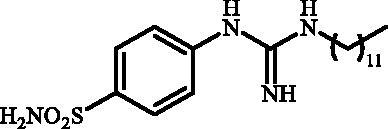


To a solution of **6** (300 mg, 1.22 mmol) in DMSO (8 ml), dodecan-1-amine (1.809 g, 9.76 mmol) was added and the reaction mixture was stirred in sealed tube at 130 °C for 2 h. After cooling to room temperature water (40 ml) was added and mixture was extracted with EtOAc (3 × 20 ml). The organic phase was dried over Na_2_SO_4_ and the solvent was removed under reduced pressure. The residue was dissolved in *i*PrOH (5 ml) under gentle warming and product was precipitated by addition of hexanes (40 ml). The precipitate formed was collected by filtration and dried in vacuum to afford **7m** (196 mg, 42%) as white powder.

^1^H NMR (300 MHz, DMSO-d_6 _+D_2_O) *δ* = 0.88 (t, 3H, *J* = 6.3 Hz), 1.15–127 (m, 18H), 1.47 (s, 2H), 3.14 (t, 2H, *J =* 6.3 Hz), 6.88 (d, 2H, *J =* 8.1 Hz), 7.61 (d, 2H, *J =* 8.1 Hz) ppm ^13^C NMR (75 MHz, DMSO-d_6 _+D_2_O) *δ* = 15.1, 23.3, 27.7, 29.9, 30.0, 30.2 (br), 30.4, 32.5, 41.5, 123.7, 127.9, 135.5, 153.4, 156.1 ppm HRMS (ESI) [M + H]^+^: *m*/*z* calcd for (C_19_H_35_N_4_O_2_S) 383.2481. Found 383.2490.

#### 2.2.16. Synthesis of 4-(3-hexadecylguanidino)benzenesulfonamide (7n)


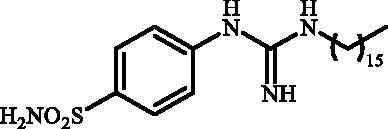


To a solution of **6** (300 mg, 1.22 mmol) in DMSO (8 ml), hexadecan-1-amine (2.356 g, 9.76 mmol) was added and the reaction mixture was stirred in sealed tube at 130 °C for 2 h. After cooling to room temperature, it was filtered through the sintered glass crucible. Water (40 ml) was added to the filtrate and the mixture was extracted with EtOAc (3 × 20 ml). The organic phase was dried over Na_2_SO_4_ and the solvent was removed under reduced pressure. The residue was washed with CHCl_3_ (50 ml) and dried under vacuum to afford **7n** (221 mg, 41%) as white powder.

^1^H NMR (300 MHz, DMSO-d_6 _+D_2_O) *δ* = 0.84 (br. s, 3H), 1.23 (br. s, 28H), 3.09 (br. s, 2H), 6.88 (br. s, 2H), 7.61 (br. s, 2H) ppm ^13 ^C NMR (75 MHz, DMSO-d_6_+ D_2_O) *δ* = 15.2, 23.6, 28.0, 30.6 (br), 32.8, 41.9, 124.0, 128.2, 135.9, 153.8, 155.9 ppm HRMS (ESI) [M + H]^+^: *m*/*z* calcd for (C_23_H_43_N_4_O_2_S) 439.3107. Found 439.3114.

#### 2.2.17. 4-Benzyl-N-(4-sulfamoylphenyl)piperazine-1-carboximidamide (7o)


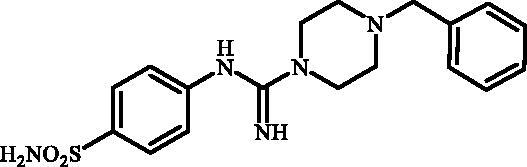


To a solution of **6** (300 mg, 1.22 mmol) in DMSO (8 ml), 1-benzylpiperazine (1.696 ml, 9.76 mmol) was added and the reaction mixture was stirred in sealed tube at 130 °C for 6 h. After cooling to room temperature water (40 ml) was added and the mixture was extracted with EtOAc (3 × 20 ml). The organic phase was dried over Na_2_SO_4_ and the solvent was removed under reduced pressure. The residual oily solid was washed with Et_2_O (20 ml) and dried under vacuum to afford **7o** (402 mg, 88%) as white powder.

^1^H NMR (300 MHz, DMSO-d_6 _+D_2_O) *δ* = 2.41 (s, 4H), 3.38 (s, 4H), 3.52 (s, 2H), 6.86 (d, 2H, *J =* 8.3 Hz), 7.28-7.39 (m, 5H), 7.65 (d, 2H, *J =* 8.3 Hz) ppm ^13 ^C NMR (75 MHz, DMSO-d_6 _+D_2_O) *δ* = 45.9, 53.5, 63.3, 123.7, 128.1, 128.2, 129.4, 130.2, 135.9, 139.0, 154.2, 156.1 ppm HRMS (ESI) [M + H]^+^: *m*/*z* calcd for (C_18_H_24_N_5_O_2_S) 374.1651. Found 374.1649.

### 2.3. CA inhibitory assay

An applied photophysics stopped-flow instrument has been used for assaying the CA-catalysed CO_2_ hydration activity^43^. Phenol red (at a concentration of 0.2 mM) was used as indicator, working at the absorbance maximum of 557 nm, with 20 mM Hepes (pH 7.5) as buffer and 20 mM Na_2_SO_4_ (for maintaining constant the ionic strength), following the initial rates of the CA-catalysed CO_2_ hydration reaction for a period of 10 − 100 s. The CO_2_ concentrations ranged from 1.7 to 17 mM for the determination of the kinetic parameters and inhibition constants. For each inhibitor, at least six traces of the initial 5 − 10% of the reaction have been used for determining the initial velocity. The uncatalysed rates were determined in the same manner and subtracted from the total observed rates. Stock solutions of inhibitor (0.1 mM) were prepared in distilled-deionised water, and dilutions up to 0.01 nM were done thereafter with the assay buffer. Inhibitor and enzyme solutions were preincubated together for 6 h at room temperature prior to assay in order to allow for the formation of the E-I complex. The inhibition constants were obtained by nonlinear least-squares methods using PRISM 3 and the Cheng-Prusoff equation, as reported earlier^44–53^, and represent the mean from at least three different determinations. All CA isoforms were recombinant ones obtained in-house as reported earlier^54–57^.

## 3. Results and discussion

### 3.1. Chemistry

**Scheme 1.**
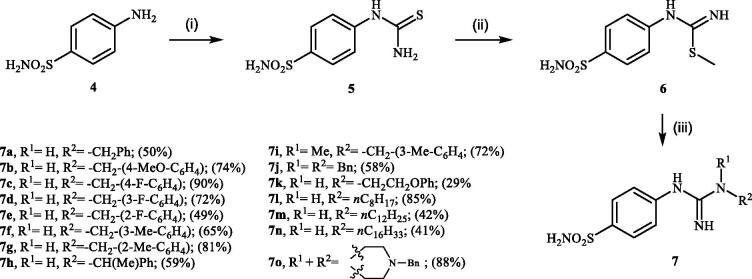
Reagents and conditions: (i) KSCN, aq. 3.5 M HCl, reflux, 3 h, 31%; (ii) MeI, DMF, 40 °C, 2.5 h, 70%; (iii) HNR^1^R^2^ (8 equiv.), DMSO, 130 °C, 2–6 h.

Desired 4–(3-alkyl/benzyl-guanidino)benzenesulfonamides **7a–o** were obtained in three step synthesis (Scheme 1). In the first step 4-aminobenzenesulfonamide (**4**) was reacted with KSCN under acidic conditions, thus obtaining intermediate-4-thioureidobenzenesulfonamide (**5**). In the intermediate **5** the reactive thiourea functionality was selectively converted into methyl carbamimidothioate **6** through the treatment with methyl iodide in DMF in the absence of base or catalyst. In the subsequent reaction methyl carbamimidothioate **6** was reacted various primary and secondary aliphatic and benzylic amines in DMF at elevated temperature, affording the desired 4-(3-alkyl/benzyl-guanidino)benzenesulfonamides **7** in satisfying to high yields ranging from 29% to 90%.

### 3.2. Carbonic anhydrase inhibition

The obtained series of 4–(3-alkyl/benzyl-guanidino)benzenesulfonamides **7a–o** were investigated for their CA inhibitory properties by using a stopped-flow CO_2_ hydrase assay^43^ and three human CA isoforms (hCA I, II and VII) known to be drug targets for neurological conditions^37^^,^^58–62^ (Table 1).

**Table 1.** Inhibition data of human CA isoforms I, II and VII using **AAZ** as standard drug. 
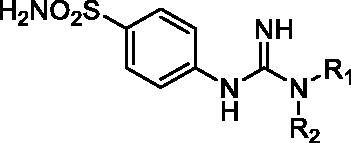

Compound	R_1_	R_2_	*K_i_* (nM)^a^		
			CA I	CA II	CA VII
**7a**	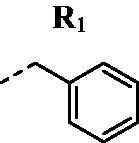	H	746.5	7.7	10.1
**7b**	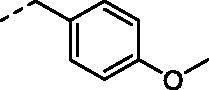	H	698.7	17.7	25.1
**7c**	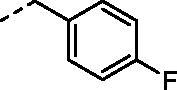	H	989.1	59.1	15.0
**7d**	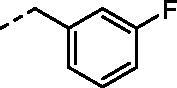	H	781.7	1.6	60.7
**7e**	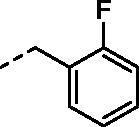	H	2988.8	3.2	44.9
**7f**	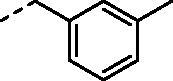	H	2932.2	0.3	0.9
**7g**	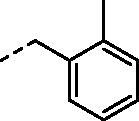	H	3163.0	0.07	0.4
**7h**	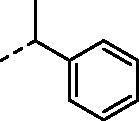	H	1319.0	9.6	1.1
**7i**	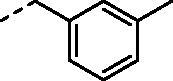	CH_3_	1137.7	0.08	0.7
**7j**	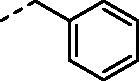	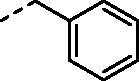	5845.5	0.6	3.2
**7k**	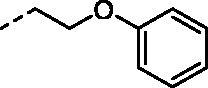	H	4183.3	6.9	8.7
**7l**	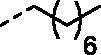	H	729.8	0.9	2.2
**7m**	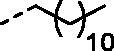	H	930.3	0.4	0.2
**7n**	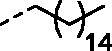	H	2154.0	0.06	0.08
**7o**	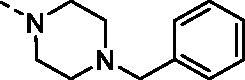		3676.8	0.1	0.06
**AAZ**	–	–	250.0	12.0	2.5

^a^Mean from three different assays, by a stopped-flow technique (errors were in the range of ±5–10% of the reported values).

As seen from data of Table 1, benzenesulfonamides **7a–o** did not significantly inhibit the cytosolic isoforms hCA I, which is considered as being an off-target isoform in our study. The ubiquitous hCA II was significantly inhibited by many benzenesulfonamides **7** studied here. Compounds **7a–e**, **7h** and **7k** had K_I_s ranging from 1.6 to 59.1 nM, in most cased being lower or comparable to those of the non-selective CA inhibitor acetazolamide (**AAZ**), which has a K_I_ of 12 nM. The rest of the derivatives **7** strongly inhibited CA II, with *K*_I_ values in the low nanomolar or subnanomolar range. Neuropathic pain associated CA VII was also effectively inhibited by most of the sulfonamides **7**, even in subnanomolar range for compounds **7f**, **7g**, **7i** and **7m–o**. However, compounds **7c**, **7h**, **7m** and **7o** (nanomolar and subnanomolar inhibitors) also exhibited remarkable selectivity towards CA VII compared to the off-target isoforms CA I and CA II.

The selectivity indexes for the inhibition of hCA VII over hCA I and II for the new compounds reported here are shown in Table 2.

**Table 2.** Selectivity indexes for hCA VII over hCA I and II inhibition with compounds **7a–7o** and acetazolamide as standard inhibitor.

	Selectivity index^a^	
Compound	hCA VII/hCA I	hCA VII/hCA II
**7°**	73.9	0.76
**7b**	27.8	0.70
**7c**	65.9	3.94
**7d**	12.8	0.02
**7e**	66.5	0.07
**7f**	3257	0.33
**7g**	7907	0.17
**7h**	1199	8.72
**7i**	1625	0.11
**7j**	1826	0.18
**7k**	480	0.79
**7l**	331	0.41
**7m**	4651	2.00
**7n**	26,925	0.75
**7o**	61,300	1.66
**AAZ**	100	4.80

^a^Calculated as the ratio K_I_(CA I or II)/K_I_(CA VII).

It may be seen that all new compounds **7a–7o** were highly selective for the inhibition of CA VII over CA I, with selectivity indexes in the range of 12.8 − 61300. On the other hand, only compounds **7c, 7h, 7m** and **7o** showed selectivity for inhibiting CA VII over CA II, with selectivity indexes of 1.66 − 8.72. Many of these new sulfonamides (e.g. **7d, 7e, 7g, 7i** and **7j**) were in fact highly hCA II selective inhibitors.

## 4. Conclusion

A series of novel 4-(3-alkyl/benzyl-guanidino)benzenesulfonamide derivatives with various long alkyl chains and functional groups on benzyl moieties through the direct catalyst-free desulfidative amination of easily accessible methyl (4-sulfamoylphenyl)carbamimidothioate with respective primary and secondary amines were obtained. The new derivatives were assayed as inhibitors of the zinc metalloenzyme CA. Three pharmacologically relevant human (h) isoforms (CA I, CA II and CA VII) were investigated. No significant inhibition of hCA I was observed, whereas some of the new derivatives were effective, low nanomolar or even subnanomolar hCA II and CA VII inhibitors. Four novel sulfonamide derivatives **7c**, **7h**, **7m** and **7o** having low nanomolar or subnanomolar *K*_I_ values and significant selectivity towards neuropathic pain related CA VII have a potential for further investigation as potential neuropathic pain attenuation agents.

## References

[1] (a) Supuran CT. Novel carbonic anhydrase inhibitors. Future Med Chem 2021;13:1568–7. 10.4155/fmc-2021-0222 34498952

[2] Supuran CT. Carbonic anhydrases: novel therapeutic applications for inhibitors and activators. Nat Rev Drug Discov 2008;7:168–81. 18167490 10.1038/nrd2467

[3] Supuran CT, Scozzafava A. Carbonic anhydrases as targets for medicinal chemistry. Bioorg Med Chem 2007;15:4336–50. 17475500 10.1016/j.bmc.2007.04.020

[4] Supuran CT, Scozzafava A, Casini A. Carbonic anhydrase inhibitors. Med Res Rev 2003;23:146–89. 12500287 10.1002/med.10025

[5] Supuran CT. Emerging role of carbonic anhydrase inhibitors. Clin Sci (Lond) 2021 May 28;135:1233–49. 34013961 10.1042/CS20210040

[6] Alterio V, Di Fiore A, D’Ambrosio K, et al. Multiple binding modes of inhibitors to carbonic anhydrases: how to design specific drugs targeting 15 different isoforms? Chem Rev 2012;112:4421–68. 22607219 10.1021/cr200176r

[7] Supuran CT. Structure and function of carbonic anhydrases. Biochem J 2016;473:2023–32. 27407171 10.1042/BCJ20160115

[8] Ivanova J, Balode A, Žalubovskis R, et al. 5-Substituted-benzylsulfanyl-thiophene-2-sulfonamides with effective carbonic anhydrase inhibitory activity: solution and crystallographic investigations. Bioorg Med Chem 2017;25:857–63. 28024887 10.1016/j.bmc.2016.11.045

[9] Alterio V, Tanc M, Ivanova J, et al. Supuran X-ray crystallographic and kinetic investigations of 6-sulfamoyl-saccharin as a carbonic anhydrase inhibitor. Org Biomol Chem 2015; 13:4064–69. 25733161 10.1039/c4ob02648a

[10] Supuran CT. How many carbonic anhydrase inhibition mechanisms exist? J Enzyme Inhib Med Chem 2016;31:345–60. 26619898 10.3109/14756366.2015.1122001

[11] Nocentini A, Supuran CT. Advances in the structural annotation of human carbonic anhydrases and impact on future drug discovery. Expert Opin Drug Discov 2019;14:1175–97. 31436118 10.1080/17460441.2019.1651289

[12] Supuran CT. Advances in structure-based drug discovery of carbonic anhydrase inhibitors. Expert Opin Drug Discov 2017;12:61–88. 27783541 10.1080/17460441.2017.1253677

[13] De Simone G, Supuran CT. (In)organic anions as carbonic anhydrase inhibitors. J Inorg Biochem 2012;111:117–29. 22192857 10.1016/j.jinorgbio.2011.11.017

[14] Pustenko A, Stepanovs D, Žalubovskis R, et al. 3H-1,2-benzoxathiepine 2,2-dioxides: a new class of isoform-selective carbonic anhydrase inhibitors. J Enzyme Inhib Med Chem 2017;32:767–75. 28537099 10.1080/14756366.2017.1316720 PMC6445229

[15] Pustenko A, Nocentini A, Balašova A, et al. Aryl derivatives of 3H-1,2-benzoxathiepine 2,2-dioxide as carbonic anhydrase inhibitors. J Enzyme Inhib Med Chem 2020;35:245–54. 31790605 10.1080/14756366.2019.1695795 PMC6896485

[16] Tars K, Vullo D, Kazaks A, et al. Sulfocoumarins (1,2-benzoxathiine-2,2-dioxides): a class of potent and isoform-selective inhibitors of tumor-associated carbonic anhydrases. J Med Chem 2013;56:293–300. 23241068 10.1021/jm301625s

[17] Tanc M, Carta F, Bozdag M, et al. 7-Substituted-sulfocoumarins are isoform-selective, potent carbonic anhydrase II inhibitors. Bioorg Med Chem 2013;21:4502–10. 23769167 10.1016/j.bmc.2013.05.032

[18] Nocentini A, Ceruso M, Carta F, Supuran CT. 7-Aryl-triazolyl-substituted sulfocoumarins are potent, selective inhibitors of the tumor-associated carbonic anhydrase IX and XII. J Enzyme Inhib Med Chem 2016;31:1226–33. 26681367 10.3109/14756366.2015.1115401

[19] Grandane A, Tanc M, Mannelli LDC, et al. 6-Substituted sulfocoumarins are selective carbonic anhdydrase IX and XII inhibitors with significant cytotoxicity against colorectal cancer cells. J Med Chem 2015;58:3975–83. 25875209 10.1021/acs.jmedchem.5b00523

[20] Maresca A, Temperini C, Vu H, et al. Non-zinc mediated inhibition of carbonic anhydrases: coumarins are a new class of suicide inhibitors. J Am Chem Soc 2009;131:3057–62. 19206230 10.1021/ja809683v

[21] Maresca A, Temperini C, Pochet L, et al. Deciphering the mechanism of carbonic anhydrase inhibition with coumarins and thiocoumarins. J Med Chem 2010;53:335–44. 19911821 10.1021/jm901287j

[22] Temperini C, Innocenti A, Scozzafava A, et al. The coumarin-binding site in carbonic anhydrase accommodates structurally diverse inhibitors: the antiepileptic lacosamide as an example and lead molecule for novel classes of carbonic anhydrase inhibitors. J Med Chem 2010;53:850–4. 20028100 10.1021/jm901524f

[23] Touisni N, Maresca A, McDonald PC, et al. Glycosyl coumarin carbonic anhydrase IX and XII inhibitors strongly attenuate the growth of primary breast tumors. J Med Chem 2011;54:8271–7. 22077347 10.1021/jm200983e

[24] Zengin Kurt B, Sonmez F, Durdagi S, et al. Synthesis, biological activity and multiscale molecular modeling studies for coumaryl-carboxamide derivatives as selective carbonic anhydrase IX inhibitors. J Enzyme Inhib Med Chem 2017;32:1042–52. 28776440 10.1080/14756366.2017.1354857 PMC6009903

[25] Maresca A, Scozzafava A, Supuran C7. 7,8-disubstituted- but not 6,7-disubstituted coumarins selectively inhibit the transmembrane, tumor-associated carbonic anhydrase isoforms IX and XII over the cytosolic ones I and II in the low nanomolar/subnanomolar range. Bioorg Med Chem Lett 2010;20:7255–8. 21067924 10.1016/j.bmcl.2010.10.094

[26] Maresca A, Supuran CT. Coumarins incorporating hydroxy- and chloro-moieties selectively inhibit the transmembrane, tumor-associated carbonic anhydrase isoforms IX and XII over the cytosolic ones I and II. Bioorg Med Chem Lett 2010;20:4511–4. 20580555 10.1016/j.bmcl.2010.06.040

[27] Grandāne A, Nocentini A, Domračeva I, et al. Development of oxathiino[6,5-b]pyridine 2,2-dioxide derivatives as selective inhibitors of tumor-related carbonic anhydrases IX and XII. Eur J Med Chem 2020; 200:112300. 32460112 10.1016/j.ejmech.2020.112300

[28] Grandane A, Nocentini A, Werner T, et al. Benzoxepinones: A new isoform-selective class of tumor associated carbonic anhydrase inhibitors. Bioorg Med Chem 2020;28:115496. 32327349 10.1016/j.bmc.2020.115496

[29] (a) Krasavin M, Sharonova T, Sharoyko V, et al. Combining carbonic anhydrase and thioredoxin reductase inhibitory motifs within a single molecule dramatically increases its cytotoxicity. J Enzyme Inhib Med Chem 2020;35:665–71. 32131646 10.1080/14756366.2020.1734800 PMC7067156

[30] (a) Podolski-Renić A, Dinić J, Stanković T, et al. Sulfocoumarins, specific carbonic anhydrase IX and XII inhibitors, interact with cancer multidrug resistant phenotype through pH regulation and reverse P-glycoprotein mediated resistance. Eur J Pharm Sci 2019;138:105012. 31330259 10.1016/j.ejps.2019.105012

[31] (a) Bonardi A, Bua S, Combs J, et al. The three-tails approach as a new strategy to improve selectivity of action of sulphonamide inhibitors against tumour-associated carbonic anhydrase IX and XII. J Enzyme Inhib Med Chem 2022;37:930–39. 35306936 10.1080/14756366.2022.2053526 PMC8942523

[32] (a) Banoglu E, Ercanlı T, Gür Maz T, et al. Series of thiadiazolyl-benzenesulfonamides incorporating an aromatic tail as isoform-selective, potent carbonic anhydrase II/XII inhibitors. ChemMedChem 2022;17:e202200056. 35266325 10.1002/cmdc.202200056

[33] Colloca L, Ludman T, Bouhassira D, et al. Neuropathic pain. Nat Rev Dis Primers 2017;3:1–19. 10.1038/nrdp.2017.2 PMC5371025 28205574

[34] Murphy KL, Bethea JR, Fischer R, Neuropathic pain in multiple sclerosis – current therapeutic intervention and future treatment perspectives. Brisbane, Australia: Exon Publications; 2017:53–69. 29261265

[35] Fornasari D. Pharmacotherapy for neuropathic pain: a review. Pain Ther 2017;6:25–33. 29178034 10.1007/s40122-017-0091-4 PMC5701897

[36] Bhuniya D, Kharul RK, Hajare A, et al. Discovery and evaluation of novel FAAH inhibitors in neuropathic pain model. Bioorg Med Chem Lett 2019;29:238–43. 30503633 10.1016/j.bmcl.2018.11.048

[37] Supuran CT. Carbonic anhydrase inhibition and the management of neuropathic pain. Expert Rev Neurother 2016;16:961–8. 27211329 10.1080/14737175.2016.1193009

[38] (a) Supuran CT. Carbonic anhydrase inhibitors. Bioorg Med Chem Lett 2010;20:3467–74. 20529676 10.1016/j.bmcl.2010.05.009

[39] Żołnowska B, Sławiński J, Pogorzelska A, et al. Carbonic anhydrase inhibitors. Synthesis, and molecular structure of novel series *N*-substituted N'-(2-arylmethylthio-4-chloro-5-methylbenzenesulfonyl)guanidines and their inhibition of human cytosolic isozymes I and II and the transmembrane tumor-associated isozymes IX and XII. Eur J Med Chem 2014;71:135–47. 24291567 10.1016/j.ejmech.2013.10.081

[40] Barresi E, Salerno S, Marini AM, et al. Sulfonamides incorporating heteropolycyclic scaffolds show potent inhibitory action against carbonic anhydrase isoforms I, II, IX and XII. Bioorg Med Chem 2016;24:921–7. 26796953 10.1016/j.bmc.2016.01.018

[41] Żołnowska B, Sławiński J, Szafrański K, et al. Novel 2-(2-arylmethylthio-4-chloro-5-methylbenzenesulfonyl)-1-(1,3,5-triazin-2-ylamino)guanidine derivatives: Inhibition of human carbonic anhydrase cytosolic isozymes I and II and the transmembrane tumor-associated isozymes IX and XII, anticancer activity, and molecular modeling studies. Eur J Med Chem 2018;143:1931–41. 29146134 10.1016/j.ejmech.2017.11.005

[42] Leitans J, Kazaks A, Balode A, et al. Efficient expression and crystallization system of cancer-associated carbonic anhydrase isoform IX. J Med Chem 2015;58:9004–9. 26522624 10.1021/acs.jmedchem.5b01343

[43] Khalifah RG. The carbon dioxide hydration activity of carbonic anhydrase. I. Stop-flow kinetic studies on the native human isoenzymes B and C. J Biol Chem 1971;246:2561–73. 4994926

[44] Vermelho AB, da Silva Cardoso V, Ricci Junior E, et al. Nanoemulsions of sulfonamide carbonic anhydrase inhibitors strongly inhibit the growth of *Trypanosoma cruzi*. J Enzyme Inhib Med Chem 2018;33:139–46. 29192555 10.1080/14756366.2017.1405264 PMC7011998

[45] Nocentini A, Carta F, Tanc M, et al. Deciphering the mechanism of human carbonic anhydrases inhibition with sulfocoumarins: computational and experimental studies. Chemistry 2018;24:7840–4. 29603439 10.1002/chem.201800941

[46] Angeli A, Carta F, Nocentini A, et al. Response to perspectives on the classical enzyme carbonic anhydrase and the search for inhibitors. Biophys J 2021;120:178–81. 33296668 10.1016/j.bpj.2020.11.011 PMC7820713

[47] Bua S, Bozdag M, Del Prete S, et al. Mono- and di-thiocarbamate inhibition studies of the δ-carbonic anhydrase TweCAδ from the marine diatom *Thalassiosira weissflogii*. J Enzyme Inhib Med Chem 2018;33:707–13. 29577755 10.1080/14756366.2018.1450400 PMC6010021

[48] Ferraroni M, Gaspari R, Scozzafava A, et al. Dioxygen, an unexpected carbonic anhydrase ligand. J Enzyme Inhib Med Chem 2018;33:999–1005. 29806484 10.1080/14756366.2018.1475371 PMC6010096

[49] Pustenko A, Nocentini A, Gratteri P, et al. The antibiotic furagin and its derivatives are isoform-selective human carbonic anhydrase inhibitors. J Enzyme Inhib Med Chem 2020;35:1011–20. 32297543 10.1080/14756366.2020.1752201 PMC7178874

[50] Akocak S, Lolak N, Bua S, Supuran CT. Discovery of novel 1,3-diaryltriazene sulfonamides as carbonic anhydrase I, II, VII, and IX inhibitors. J Enzyme Inhib Med Chem 2018;33:1575–80. 30296852 10.1080/14756366.2018.1515933 PMC6179046

[51] Nocentini A, Bonardi A, Gratteri P, et al. Steroids interfere with human carbonic anhydrase activity by using alternative binding mechanisms. J Enzyme Inhib Med Chem 2018;33:1453–9. 30221552 10.1080/14756366.2018.1512597 PMC7011995

[52] Nocentini A, Trallori E, Singh S, et al. 4-Hydroxy-3-nitro-5-ureido-benzenesulfonamides selectively target the tumor-associated carbonic anhydrase isoforms IX and XII showing hypoxia-enhanced antiproliferative profiles. J Med Chem 2018;61:10860–74. 30433782 10.1021/acs.jmedchem.8b01504

[53] Chohan ZH, Munawar A, Supuran CT. Transition metal ion complexes of Schiff-bases. Synthesis, characterization and antibacterial properties. Met Based Drugs 2001;8:137–43. 18475987 10.1155/MBD.2001.137 PMC2365267

[54] Ivanova J, Carta F, Vullo D, et al. N-Substituted and ring opened saccharin derivatives selectively inhibit transmembrane, tumor-associated carbonic anhydrases IX and XII. Bioorg Med Chem 2017;25:3583–9. 28416101 10.1016/j.bmc.2017.04.007

[55] (a) Supuran CT. Carbon- versus sulphur-based zinc binding groups for carbonic anhydrase inhibitors? J Enzyme Inhib Med Chem 2018;33:485–95. 29390912 10.1080/14756366.2018.1428572 PMC6009921

[56] (a) Pastorekova S, Casini A, Scozzafava A, et al. Carbonic anhydrase inhibitors: the first selective, membrane-impermeant inhibitors targeting the tumor-associated isozyme IX. Bioorg Med Chem Lett 2004;14:869–73. 15012984 10.1016/j.bmcl.2003.12.029

[57] (a) Sarikaya SB, Gülçin I, Supuran CT. Carbonic anhydrase inhibitors: Inhibition of human erythrocyte isozymes I and II with a series of phenolic acids. Chem Biol Drug Des 2010 May;75:515–20. 20486938 10.1111/j.1747-0285.2010.00965.x

[58] (a) Di Cesare Mannelli L, Micheli L, Carta F, et al. Carbonic anhydrase inhibition for the management of cerebral ischemia: in vivo evaluation of sulfonamide and coumarin inhibitors. J Enzyme Inhib Med Chem 2016;31:894–99. 26607399 10.3109/14756366.2015.1113407

[59] Angeli A, Carta F, Nocentini A, et al. Carbonic anhydrase inhibitors targeting metabolism and tumor microenvironment. Metabolites 2020;10:412. 10.3390/metabo10100412 PMC7602163 33066524

[60] Nocentini A, Angeli A, Carta F, et al. Reconsidering anion inhibitors in the general context of drug design studies of modulators of activity of the classical enzyme carbonic anhydrase. J Enzyme Inhib Med Chem 2021;36:561–80. 33615947 10.1080/14756366.2021.1882453 PMC7901698

[61] Abdoli M, Angeli A, Bozdag M, et al. Synthesis and carbonic anhydrase I, II, VII, and IX inhibition studies with a series of benzo[d]thiazole-5- and 6-sulfonamides. J Enzyme Inhib Med Chem 2017;32:1071–8. 28753093 10.1080/14756366.2017.1356295 PMC6010138

[62] Abdoli M, Bozdag M, Angeli A, et al. Benzamide-4-sulfonamides are effective human carbonic anhydrase i, ii, vii, and ix inhibitors. Metabolites 2018;8:37. 10.3390/metabo8020037 PMC6027465 29857578

